# Impact of a structured referral algorithm on the ability to monitor adherence to appropriate use criteria for transthoracic echocardiography

**DOI:** 10.1186/s12947-016-0075-2

**Published:** 2016-08-15

**Authors:** Steven Promislow, Joseph G. Abunassar, Behnam Banihashemi, Benjamin J. Chow, Girish Dwivedi, Kasra Maftoon, Ian G. Burwash

**Affiliations:** Division of Cardiology, Department of Medicine, University of Ottawa Heart Institute, University of Ottawa, 40 Ruskin Street, Ottawa, ON K1Y 4W7 Canada

**Keywords:** Transthoracic echocardiography, Appropriate use criteria, Diagnostic requisitions, Quality improvement

## Abstract

**Background:**

Many free-form-text referral requisitions for transthoracic echocardiography (TTE) provide insufficient information to adequately evaluate their adherence to Appropriate Use Criteria (AUC). We developed a structured referral requisition algorithm based on requisition deficiencies identified retrospectively in a derivation cohort of 1303 TTE referrals and evaluated the performance of the algorithm in a consecutive series of cardiology outpatient referrals.

**Methods:**

The validation cohort comprised 286 consecutive TTE outpatient cardiology referrals over a 2-week period. The relevant AUC indication was identified from information extracted from the free-form-text requisition. The structured referral algorithm was applied prospectively to the same cohort using information from the free-form-text requisition, electronic medical record and ordering clinicians. Referrals were classified as appropriate, uncertain, non-adherent (inappropriate) or unclassifiable based on the American College of Cardiology Foundation 2011 AUC.

**Results:**

Only 28.7 % of free-form-text requisitions provided adequate information to identify the relevant AUC indication, as compared to 94.4 % of referrals using the structured referral algorithm (*p* < 0.001). The structured algorithm improved identification in the AUC categories of general evaluation of cardiac structure/function (100 % vs. 43.0 %, *p* < 0.001); valvular function (100 % vs. 23.0 %, *p* < 0.001); hypertension, heart failure or cardiomyopathy (100 % vs. 20.3 %, *p* < 0.001); and adult congenital heart disease (100 % vs. 0 %, *p* < 0.001). By applying the algorithm, the number of identifiable non-adherent studies increased from 2.6 to 10.4 % (*p* <0.001).

**Conclusions:**

Use of a structured TTE referral algorithm, as opposed to a free-form-text requisition, allowed the vast majority of referrals to be monitored for AUC adherence and facilitated the identification of potentially inappropriate referrals.

## Background

The last 15 years have seen significant increases in health care expenditures leading to concerns about the sustainability of such growth [1, 2]. Cardiac diagnostic imaging represents a large component of the increase in health care expenditures, and the use of echocardiography, in particular, continues to rise [2–5]. There have been significant recent efforts to ensure that the use of diagnostic imaging is clinically indicated and of value. The American College of Cardiology Foundation (ACCF) Task Force has developed guidelines for the appropriate use of cardiac diagnostic services [6–9]. The first set of Appropriate Use Criteria (AUC) for echocardiography were published in 2007 [9] and later updated in 2011 [6]. These criteria have been incorporated into educational efforts directed at physicians and patients in an attempt to limit unnecessary tests, treatments and procedures, such as the Choosing Wisely campaign, introduced in the United States in 2012 and in Canada in 2014 [10]. While the rate of increase in echocardiography use has slowed in recent years in association with the development of AUC and a reduction in reimbursement for echocardiography, the proportion of US Medicare beneficiaries receiving echocardiography services in 2011 exceeded those in 2007 when the AUC guidelines were published, and the average number of echocardiography studies per recipient per annum has continued to rise [4, 11]. It is estimated that approximately $750 billion of health care spending per year in the United States is wasted, with overuse of services beyond evidence-established levels playing a significant role [12].

Implementing AUC in echocardiography can have a direct clinical impact as appropriate studies are more likely to reveal new and major findings and are more likely to result in a patient care intervention [13]. Unfortunately ~10 % of transthoracic echocardiography (TTE) referrals are inappropriate or non-adherent to AUC [13, 14]. The prevalence of non-adherent referrals tends to be greater in outpatients [14–17], community settings [18] and non-specialist referrals [15, 19]; however inappropriate referrals have been reported in all clinical settings [13–20]. Therefore, adherence to AUC is important for cost containment, effective resource utilization and best practice clinical medicine.

Although the clinician orders the echocardiogram, the onus for the application of AUC has in part become the responsibility of the echocardiography laboratory. Demonstration of AUC monitoring and adherence is a requirement for the accreditation of echocardiography laboratories by government insurers, provincial regulatory bodies and international accreditation organizations such as the Intersocietal Accreditation Commission (IAC) [21, 22]. Unfortunately, monitoring adherence to AUC can be challenging. Clinicians usually request echocardiograms by completing free-form-text requisitions, whether in an electronic or paper format. In a recent study at our institution, of 1303 consecutive TTE referrals, more than 26 % of requisitions did not provide enough information to determine if the referral met AUC [17]. Further, 41 % of requisitions from cardiologists provided insufficient information [17]. Banihashemi and colleagues concluded that structured requisition formats that required referring physicians to provide AUC-relevant information were needed to facilitate the monitoring and application of AUC in the echocardiography laboratory.

Using information from this study [17], we designed a structured TTE referral requisition algorithm with mandatory fields that included the most common inadequacies identified, along with components of the ACCF 2011 AUC. The objective of this study was to evaluate the performance of the structured TTE referral algorithm compared to the standard free-form text referral requisition to evaluate AUC adherence in a consecutive series of cardiology outpatient referrals over a 2 week period.

## Methods

### Echocardiography laboratory

The University of Ottawa Heart Institute (UOHI) echocardiography laboratory provides outpatient and inpatient echocardiography services for both community and hospital based primary care physicians and specialists and is staffed by seven full-time Level 3 echocardiographers. The laboratory is accredited by IAC and has an annual TTE volume of 18,934 studies (2015).

### Structured requisition algorithm

In our previous study of 1303 consecutive echocardiogram requisitions, 26.2 % of requisitions contained inadequate information to determine whether the referral was adherent to AUC [17]. The vast majority of inadequate requisitions occurred within the AUC categories of (1) evaluation of cardiac structure and function, (2) evaluation of valvular function, (3) evaluation of hypertension, heart failure, or cardiomyopathy and (4) adult congenital heart disease. The inadequacy related primarily to a failure of the requisition to report on a change in clinical status, provide the date of a previous echocardiogram if one had been done, or report the type and/or severity of a known valve lesion.

**Fig. 1:**
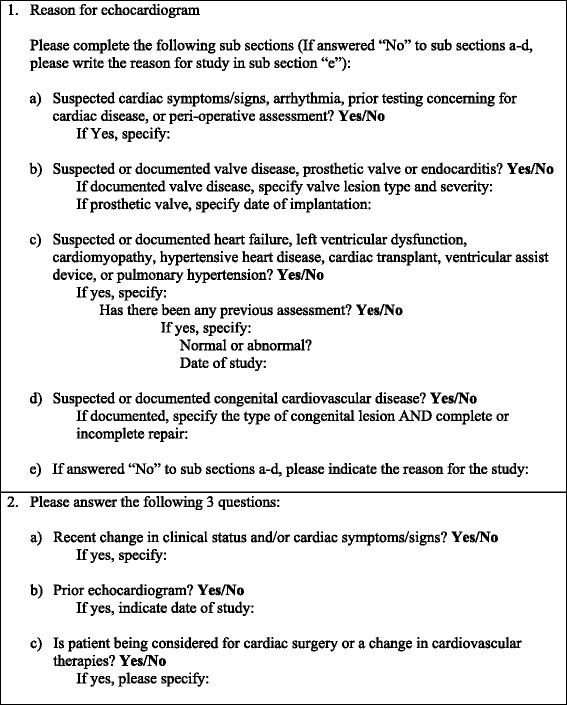
Structured algorithm for transthoracic echocardiogram requisition

Using this information, we developed a structured requisition algorithm with mandatory fields which included the most common inadequacies, along with components of the ACCF 2011 AUC (Fig 1). This was applied retrospectively to the initial derivation cohort of 1303 requisitions and was successful in classifying all but 6 requisitions based on AUC.

### Study population

The utility of the newly-designed structured referral requisition algorithm relative to the standard free-form-text requisition was assessed by applying the structured referral requisition algorithm prospectively to a validation cohort of consecutive TTE referrals from our outpatient cardiology clinic over a 2-week period (*n* = 286).

### Echocardiography requisition review protocol

The review protocol was divided into two sequential phases.

First, the 286 free-form-text referral requisitions were evaluated by two reviewers for appropriateness of indication using only information extracted from the referral requisition (see Appendix). Requisition information was matched to the relevant indication in the ACCF 2011 AUC and categorized as classifiable, if they provided adequate information to identify a specific AUC indication, or not classifiable. If the referral requisition was classifiable, it was further categorized as appropriate, uncertain or non-adherent (inappropriate) based on the ACCF 2011 AUC.

Second, the structured requisition algorithm was applied to the same 286 referral requisitions by the same two reviewers, with all of the mandatory fields populated by information known to the ordering clinician (either provided in the free-form-text requisition, taken from previous clinic notes within the electronic medical records, or acquired through direct contact with the ordering clinician when required). Each requisition was again categorized as classifiable or not using the ACCF 2011 AUC, and further categorized as appropriate, uncertain or non-adherent based on the ACCF 2011 AUC.

Identification of the relevant ACCF 2011 AUC indication during review of the free-form-text requisitions and application of the structured algorithm was performed by the consensus of two reviewers, and a third reviewer if consensus was not achieved.

### Statistical analysis

The primary outcome measure was the percent of referral requisitions that were classifiable using the free-form-text referral requisition and the structured requisition algorithm. The secondary outcome measure was the percent of echocardiograms found to be non-adherent to AUC using each approach. Comparisons were made using Fisher’s exact test, with statistical significance set at *p* <0.05.

## Results

The study cohort consisted of 286 consecutive TTE referrals received over the 2-week period. Median age was 61 years old [46–73 inter-quartile range]. 57.3 % were male. Of the 286 TTE referrals, 4 referrals were excluded because the structured referral requisition algorithm could not be used as additional medical information was not available (*n* = 2), the request was cancelled (*n* = 1), or the patient did not provide consent for chart review (*n* = 1). Of the remaining 282 TTE referrals, 268 (95.0 %) were for an indication outlined in the ACCF 2011 AUC, as determined by a complete review of the referral requisition, electronic medical record and information from the referring physician.

In those patients with a TTE referral encompassed in the ACCF 2011 AUC, only 28.7 % (*n* = 77) of the free-form-text requisitions provided adequate information to classify the referral to a specific indication in the ACCF 2011 AUC (Table 1). In 71.3 % (*n* = 191) of TTE referrals, the free-form-text requisition was unclassifiable, containing insufficient information to identify the relevant ACCF 2011 AUC indication.

**Table 1:** Distribution of ACCF 2011 AUC indication categories in classifiable referrals using a free-form-text requisition and the structured referral requisition algorithm

AUC Indication Category of Classifiable Requisitions	Free-form-text requisition *n* = 77 (%)	Structured algorithm requisition *n* = 253 (%)
General evaluation of cardiac structure and function	34 (44.2)	79 (31.2)
Cardiovascular evaluation in an acute setting	7 (9.1)	0 (0)
Evaluation of valvular function	20 (26.0)	87 (34.4)
Evaluation of intracardiac and extracardiac structures and chambers	1 (1.3)	0 (0)
Evaluation of aortic disease	1 (1.3)	0 (0)
Evaluation of hypertension, heart failure or cardiomyopathy	14 (18.2)	69 (27.3)
Adult congenital heart disease	0 (0)	18 (7.1)

When the structured referral requisition algorithm was applied to the same 268 TTE referrals encompassed in the ACCF 2011 AUC, 94.4 % (*n* = 253) of requests were classifiable, a significant improvement from the 28.7 % classifiable requisitions achieved using the free-form-text requisition (*p* <0.001) (Table 1). The structured referral requisition algorithm performed well in patients with both a classifiable and non-classifiable free-form text referral requisition. In 191 patients with a non-classifiable free-form-text requisition, application of the structured requisition algorithm resulted in 96.9 % (*n* = 185) of referrals being classifiable. The structured requisition algorithm classified 88.3 % of referrals when the free-form text requisition was classifiable. TTE referrals for ACCF 2011 AUC indications that were unclassifiable by our structured requisition algorithm (*n* = 15) are shown in Table 2. Evaluation/re-evaluation of ventricular function following ACS accounted for 80 % of unclassifiable requisitions using our structured referral requisition algorithm.

**Table 2:** Referral indications (based on ACCF 2011 AUC) that were not identified by our structured referral requisition algorithm

AUC Indication Number	Description	Number of Requisitions
24	Initial evaluation of ventricular function following ACS	2
25	Re-evaluation of ventricular function following ACS during recovery phase when results will guide therapy	10
59	Suspected pericardial conditions	1
65	Re-evaluation of known ascending aorta dilation or history of aortic dissection with a change in clinical status or cardiac exam or when findings may alter management or therapy	2

**Fig. 2:**
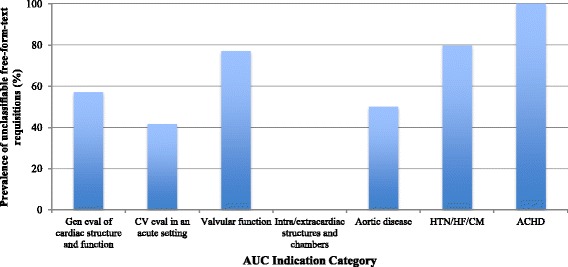
Prevalence of unclassifiable free-form-text requisitions by ACCF 2011 AUC category (*n* = 268). CV, cardiovascular; HTN, hypertension; HF, heart failure; CM, cardiomyopathy; ACHD, adult congenital heart disease

**Fig. 3:**
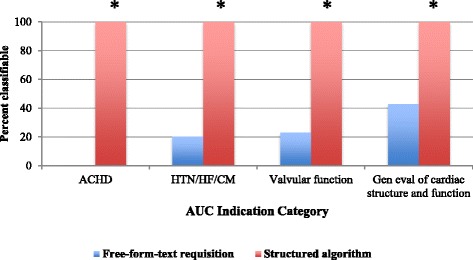
Prevalence of classifiable TTE referrals using the free-form-text requisition (*blue bars*) and structured referral requisition algorithm (*red bars*) in the four AUC categories with the largest patient numbers. * *p* <0.001 for comparison of prevalences between the structured referral requisition algorithm and free-form-text requisition

The prevalence of unclassifiable free-form-text requisitions in each ACCF 2011 AUC indication category is shown in Fig. 2. Over 50 % of free-form-text referral requisitions were unclassifiable in the categories of (1) adult congenital heart disease (100 % of requisitions unclassifiable), (2) evaluation of hypertension, heart failure or cardiomyopathy (79.7 % of requisitions unclassifiable), (3) evaluation of valvular function (77.0 % of requisitions unclassifiable), and (4) general evaluation of cardiac structure and function (57.0 % of requisitions unclassifiable). The structured algorithm improved the identification of the relevant ACCF 2011 AUC indication in all four categories such that 100 % were classifiable (*p* <0.001 for each) (Fig. 3).

**Fig. 4:**
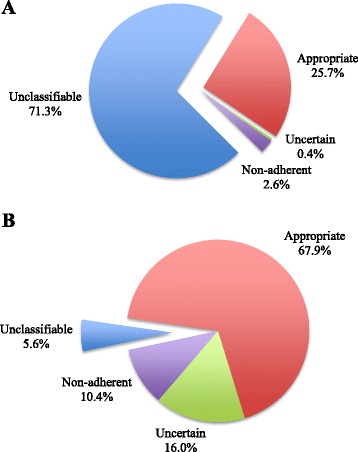
Classification of studies based on ACCF 2011 AUC using the free-form-text requisition (Fig. 4a) and structured algorithm requisition (Fig. 4b)

The prevalence of appropriate, uncertain, non-adherent (inappropriate) and unclassifiable referrals using the free-form-text requisition and structured referral requisition algorithm are shown in Fig. 4. With application of the structured referral algorithm, the number of identifiable non-adherent studies based on the ACCF 2011 AUC increased from 2.6 to 10.4 % (*p* <0.001).

## Discussion

The recently published ACCF 2011 AUC for echocardiography is a useful tool for physicians to guide their use of echocardiography, ensuring that echocardiography is used in clinical settings where it can provide the greatest diagnostic and prognostic value while also identifying scenarios where the diagnostic benefit is minimal. AUC have been adopted within the United States and other countries and serves as a quality measure during the accreditation of echocardiography laboratories by government and international organizations [21–23].

Unfortunately, most echocardiography laboratories use free-form-text referral requisitions, either in paper or electronic format, which limits the ability of an echocardiography laboratory to apply AUC to their referrals. In our study cohort, only 29 % of requisitions provided sufficient information to determine the specific AUC indication. Many physicians are not familiar with the specific information required for an AUC indication and as such may not provide the necessary information on the referral requisition for the echocardiography laboratory to determine the AUC indication and appropriateness of the referral. The most common deficiencies identified on free-form-text referrals relate to: 1) assessment of valvular heart disease, as the severity of the lesion, date of last assessment, and/or date of valve surgery (if applicable) must be provided to determine the AUC indication and appropriateness; 2) assessment of heart failure, as the date of last assessment and presence of any change in clinical condition must be provided; and 3) inadequate information about clinical status, which is applicable across multiple AUC indication categories [17]. Clearly, free-form-text referral requisitions provide a poor tool for evaluating the appropriateness of a referral.

To date, published studies evaluating the appropriateness of echocardiography have employed an extensive review of the patient’s medical record, including diagnostic requisitions, written or electronic patient charts, electronic databases and contact with the referring physicians [14, 15, 20, 24–30]. This retrospective process requires a significant investment of time and personnel by an echocardiography laboratory, is challenging without electronic access to the patient’s complete medical record, and is limited in scope since the echocardiography laboratory cannot evaluate all referrals in real-time, but rather only retrospectively sample a small portion of their volume for AUC adherence.

Our new structured referral requisition algorithm, when prospectively applied to consecutive referrals from our centre’s outpatient cardiology clinic, resulted in a dramatic reduction in unclassifiable TTE referral requisitions compared to the free-form-text requisition. The specific AUC indication could be identified in 94.4 % of TTE referrals using the structured requisition algorithm, compared to only 28.7 % using the free-form-text requisition. Importantly, the structured referral requisition algorithm improved our ability to identify non-adherent or potentially inappropriate studies; 10.4 % of requisitions were ultimately identified as non-adherent to AUC using the structured referral requisition algorithm, as compared to only 2.6 % of referrals using the free-form-text referral requisition.

Fifteen referrals (5.6 %) were not classifiable with our algorithm. This was primarily due to a failure of the algorithm to include fields for the assessment of ventricular function following an acute coronary syndrome (*n* = 12) and the assessment of aortopathy (*n* = 2). We used strict criteria and deemed a referral request unclassifiable if the reason for the referral did not clearly fall within the listed mandatory fields of our structured algorithm. While our algorithm does not specifically address ventricular function following an acute coronary syndrome, it is conceivable and likely that many physicians would describe a recent acute coronary syndrome as a recent change in clinical status and/or cardiac symptoms/signs. This would fulfill an indication field in the structured referral requisition algorithm and make the requisition classifiable, resulting in an even lower rate of unclassifiable requisitions than we have reported using our strict methodology. Nevertheless, with a further clarification and modification of the structured referral algorithm to address these two issues, all but one requisition would be classifiable.

The vast majority of echocardiography laboratories use free-form-text referral requisitions, although the requisition format may differ and may include a limited number of mandatory fields. This could impact on the prevalence of classifiable requisitions and lead to a prevalence of classifiable requisitions that differs from the 29 % observed in our cohort. However, our fully structured referral requisition algorithm avoids any requirement of the ordering physician to have knowledge of specific criteria for an AUC indication and thus has an inherent advantage over requisitions with more limited mandatory fields.

This study was conducted at a single tertiary-care academic centre, and it is possible that referral practices may differ at other institutions and could impact on the prevalence of unclassifiable requisitions. Although the structured referral algorithm was derived in a large unselected inpatient and outpatient referral population (*n* = 1303) with only 6 unclassifiable requisitions, the algorithm was prospectively tested in a cohort of consecutive TTE referrals from an outpatient cardiology clinic consisting of >30 cardiologists. In the 2010–2011 fiscal year in Ontario, Canada, 84.5 % of all echocardiograms were performed on an outpatient basis and approximately 60 % were referred from cardiologists, significantly more than any other specialty. Outpatient referrals from cardiologists have the highest rate of unclassifiable requisitions and therefore provide a suitable population to evaluate our structured referral algorithm [17]. However, caution should be taken in applying these results to other patient populations, specifically inpatients or outpatient referrals from non-cardiology practices, until these populations are more formally evaluated.

The ordering physician completed the free-form-text referral requisition; however, completion of the structured referral requisition algorithm was performed by study reviewers rather than the ordering physicians themselves. This was required to avoid introducing bias to the information provided by an ordering physician on subsequent free-form-text referral requisition submissions. While completion of the structured referral requisition algorithm by ordering physicians rather than study reviewers might affect the observed performance of our structured referral requisition algorithm, we believe the impact is likely small. First, reviewers only used information from the initial free-form-text referral requisitions, the referring physician’s notes or direct contact with the referring physician when populating the mandatory fields of the structured algorithm, information readily known to the ordering physician. Second, the questions in the structured algorithm are straightforward and minimize any ambiguity in interpretation. Third, referral requests were deemed unclassifiable by reviewers if the reason for the referral did not clearly fall within the listed mandatory fields. We believe this approach helped to eliminate any potential bias in favour of the structured referral algorithm. Nevertheless, performance of the structured referral requisition algorithm when completed by the ordering physician should be confirmed in a prospective study.

Our new structured TTE requisition shows promise in its ability to classify requisitions based on ACCF 2011 AUC that were unclassifiable using our standard free-form-text referral form. The new structured referral algorithm is relatively simple to fill out, and could be even further simplified using an electronic format in which the defaults for each question could be set to “not applicable” and only the question that applies would require further completion. Further, an electronic requisition could be programmed not to let an ordering physician submit a requisition if insufficient information is provided, or identify unclassifiable or non-adherent requisitions for further review between the referring physician and echocardiography laboratory, serving as a constraint or stop function.

The benefits of our structured referral requisition algorithm to classify previously unclassifiable requisitions are multiple. Firstly, the algorithm facilitates the process of real-time evaluation of appropriateness at the time of referral. Secondly, studies identified as non-adherent to AUC could be postponed in advance for review and cancelled if warranted, reducing both echocardiography laboratory costs and wait times. Thirdly, such a structured referral requisition algorithm would provide an opportunity for physician education as to the reason for the cancellation/postponement. Educational intervention has been demonstrated to reduce both the number of inappropriate outpatient transthoracic echocardiograms ordered by cardiology physicians-in-training [31] as well as the number of inappropriate echocardiograms on an inpatient medical service [29], and another study is ongoing looking at the benefit of a similar intervention in a more general population of physicians ordering echocardiograms [32]. If a submitted requisition is flagged as being non-adherent, further discussion with the echocardiography laboratory as needed could explain the rationale for the cancellation/postponement or conclude that the indication is actually appropriate after more information is provided. If it is found that the majority of these previously unclassifiable requisitions are in fact appropriate, then it may suggest that the main limiting factor in echocardiogram wait-times is not inappropriate referrals as some have argued [33], but rather resource availability. Correctly classifying the vast majority of referral requisitions using the structured referral requisition would provide transparency to the process of TTE referral that has yet to be achieved. It is our hope that with further investigation into different referral populations, the application of this structured referral requisition will allow echocardiography labs, accreditation organizations and government to better streamline the TTE referral process and optimize resource allocation.

## Conclusion

In a consecutive cohort of TTE referrals from an outpatient cardiology clinic, free-form-text referral requisitions provided adequate information to determine AUC adherence in only 28.7 % of referrals. In contrast, use of a structured referral requisition algorithm with mandatory fields allowed AUC adherence to be determined in 94.4 % of TTE referrals. Importantly, the structured referral requisition algorithm improved our ability to identify non-adherent TTE referrals compared to a free-form-text referral requisition. Application of a structured TTE referral algorithm by an echocardiography laboratory has the potential to provide real-time monitoring of AUC adherence.

## Abbreviations

ACCF, American College of Cardiology Foundation; AUC, appropriate use criteria; IAC, Intersocietal Accreditation Commission; TTE, transthoracic echocardiography; UOHI, University of Ottawa Heart Institute

## Figures and Tables

**Fig. 5:**
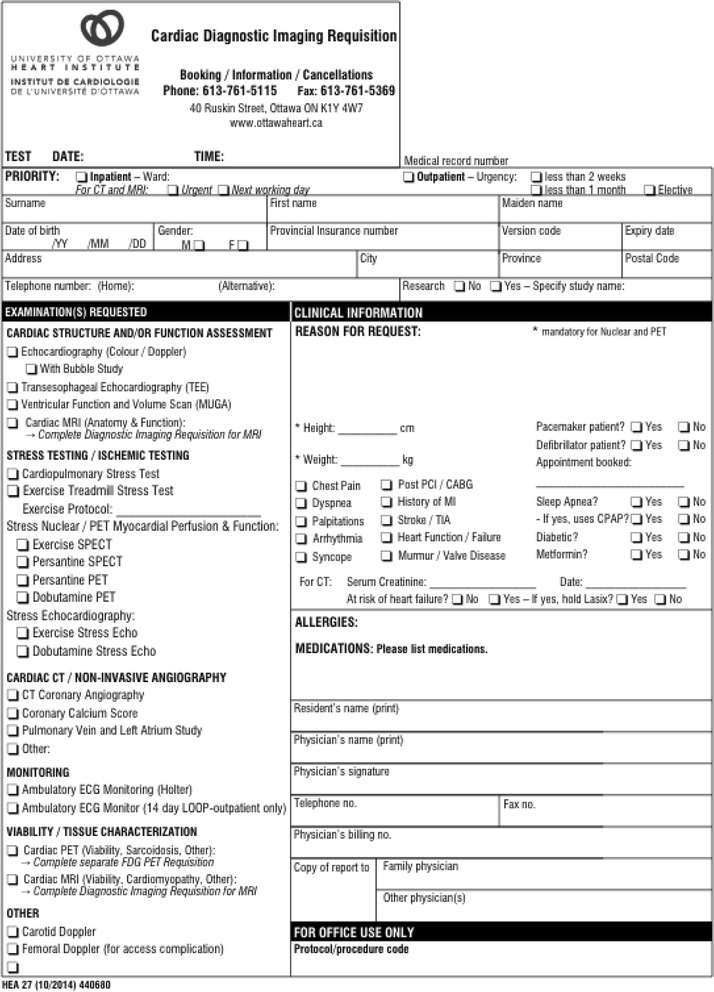
University of Ottawa Heart Institute cardiac diagnostic imaging free-form-text referral requisition
